# Screening of Potential Stress Biomarkers in Sweat Associated with Sports Training

**DOI:** 10.1186/s40798-020-00294-3

**Published:** 2021-01-22

**Authors:** Maria João Nunes, Cristina M. Cordas, José J. G. Moura, João Paulo Noronha, Luís Cobra Branco

**Affiliations:** LAQV, REQUIMTE, Departamento de Química, Faculdade de Ciências e Tecnologia, Universidade Nova de Lisboa, Campus de Caparica, 2829-516 Caparica, Portugal

**Keywords:** Biomarkers, Stress, Physical training, Sweat, LC-MSMS

## Abstract

**Background:**

Intense and continuous physical training in sports is related with psychological and physiological stress, affecting the health and well-being of athletes. The development of non-invasive sampling methodologies is essential to consider sweat as a potential biological fluid for stress biomarker assessment. In the current work, the identification in sweat samples of potential molecules that may be used as stress biomarkers was pursued.

**Methods:**

A sweat pool sample from football players after a 90-min intense training game was studied.

**Results:**

An analysis method using liquid chromatography with detection by tandem mass spectrometry (LC-MSMS) to attain a screening profile of sweat composition is presented. The major focus was on neurotransmitters (e.g. monoamines and metabolites) and other biological molecules related with physical training, such as precursors of biogenic amines (phenylaniline, tyrosine, etc.).

**Conclusions:**

This study allowed the identification of small biomolecules, neurotransmitters and other related molecules in sweat that are potentially associated with stress conditions. The developed methodology intends to contribute to the assessment and study of physical and psychological stress biomarkers related with intense sports using non-invasive methods.

**Supplementary Information:**

The online version contains supplementary material available at 10.1186/s40798-020-00294-3.

## Introduction

Athletes and other groups of people that have an intense physical training (Military, Fireman, amongst others) frequently suffer episodes of physical and/or psychological stress which may have serious consequences (increased susceptibility to inflammatory diseases such as asthma and autoimmunity) and influence on the individual behaviour and health [1]. The monitoring of stress biomarkers has been progressively recognised as an important tool due to its clear importance in medicine (e.g. precocious detection of diseases, treatments) [2, 3]. Also, in high competition sports, it is important to maximize athletes’ performance and avoid undesired secondary effects such as muscle injury and chronic inflammation (related with immunological responses) [4, 5]. There are multiple ways of detecting biomarkers in the human body, such as the analysis of the different physical parameters and by the biochemical analysis of body fluids like urine, saliva, sweat, and blood. Less invasive methods such as sweat analysis are of increasing interest for continuous health monitoring [6].

Sweat (also designated as perspiration) is produced by sweat glands that are classified into three main types: apocrine, apoeccrine, and eccrine [7]. Apocrine and apoeccrine glands are limited to certain regions of the body (e.g. the axillae region), and these do not become active until puberty [8]. Eccrine sweat glands, located across most of the body surface, are primarily responsible for thermoregulatory sweating [9, 10] by producing a fluid that is largely water (99%), salts [11] and a broad range of biological metabolites [12–15].

The ability to use sweat as an analytical fluid provides the opportunity for non-invasive sampling for early and continuous diagnosis. Depending on the analytical techniques, the sampling and preparation for analysis of sweat may be easier and faster by comparison with other biological fluids, in particular blood [16, 17]. Clinical use is currently very restricted, as there is only a single validated test to detect cystic fibrosis [12, 17, 18]. Nevertheless, sweat is indeed of increasing interest in research and has a high potential to shape clinics in the future with already several documented approaches for sweat sampling and analysis [14]. In a review article, Jadoon et al. [14] concluded that the metabolisms associated to macromolecules in sweat glands produce lower molecular weight metabolites. Also, chemical composition of perspiration varies between individuals and dietary requirements (for mineral elements) are a function of intake, absorption and losses [15]. The causes and period of sweating amongst other factors may also influence its composition (e.g. exercise or fever) [19]. Although with some intrinsic limitations, sweat is an attractive biological fluid source of chemical biomarkers mainly because it may be obtained in a much lesser invasive methodology than other fluids such as serum, and still constitutes a rich source of proteins and peptides [14]. Also, small amounts of the following substances are also present: nitrogenous compounds such as amino acids and urea; metal and non-metal ions such as potassium, sodium, and chloride; several metabolites including lactate and pyruvate; and xenobiotics such as drug molecules [11]. Athletes lose water and electrolytes because of thermoregulatory sweating during physical training, and it is well known that the rate and composition of sweat loss can vary considerably within and among individuals [19]. Research is still needed to demonstrate the benefit from the wide potential of sweat as clinical sample [18]. As so, the current study proposes to develop an analytical methodology to identify the profile of potential human stress biomarkers linked to the effect of sports training, aiming for the protection of athlete’s health and/or contributing to optimal sports performance through injury prevention [4], diagnosis or treatment [2, 3].

The aim of this work was to perform a sweat screening profile identifying small biological molecules directly or indirectly related with stress exercise training that can be detected by one LC-MSMS analytical step. The initial strategy was to define which potential target molecules may be under concern. A bibliographic research on the state of the art concerning the function of the eccrine gland [20] together with reported artificial sweat composition [16] and data of already known potential target biological molecules [11, 12, 14–16, 18, 19, 21–23] was the method to predict the molecules chemical and biological families that could be found in the screening. An example is some neurotransmitters (NTs) that were already associated with stress (e.g. 5-HT [24, 25]). Considering the chemical properties of these molecules, an analytical methodology using LC-MSMS for separation and detection was developed to accomplish the identification of these and other molecules in one analytical detection step.

The complexity of the equipment involved for sweat analysis depends on the target analyte. Due to the very low concentrations of endogenous metabolites present in sweat [21], this study focus on the development of an analytical methodology for the identification of stress biomarkers by liquid chromatography with detection by tandem mass spectrometry (LC-MSMS) aiming to get a screening profile of stress biomarkers in sweat samples composition after exercise.

## Material and Methods

### Chemicals and Reagents

#### Analytical Standards

All chemicals and reagents were of commercial origin. Analytical standard biomarkers tested:

(-)-Epinephrine (E, ≥ 99%, Sigma®), (-)-Norepinephrine (NE, ≥ 98%, Sigma-Aldrich®), L-Phenylalanine (Phe, ≥ 99%, BioUltra, Sigma®), L-Tryptophan (Trp, ≥ 98% HPLC, Sigma-Aldrich®), L-Tyrosine (Tyr, ≥ 98% HPLC, Sigma-Aldrich®), L-Histidine.HCl (His, ≥ 98% HPLC, Sigma®), L-Lysine (Lys, ≥ 95% HPLC, analytical standard, Sigma®) and L-Ascorbic acid (Asc, PHR, certified reference material, Supelco).

Analytical solvents, methanol and acetonitrile solvents for UHPLC-MS grade and Formic acid for LC-MS grade, were supplied from Carlo Erba® Reagents S.A.S.

Ultrapure water was supplied from a Milli-Q® ultrapure water system equipped at the end of assembly line with a Milli-Q® Reference and a Q-POD® element.

#### Standard Solution Preparation

Stock solutions were prepared to a concentration of 2 mg ml^-1^ in methanol and stored at − 20 °C before use. Diluted solutions of 1 ng ml^-1^ in methanol were prepared daily.

### Eccrine Sweat Sample Collection

This prospective study was conducted according to the Declaration of Helsinki, and the protocol was approved by the ethics committee of Universidade Nova de Lisboa (approval reference Parecer_CE18082020). All study subjects provided written informed consent prior to participation in the study.

Eccrine sweat samples were obtained from 10 healthy volunteers, aged between 22 and 26 years. A total of 10 sweat samples were collected from the forehead and face of the volunteers, after a 90-min football match. The healthy volunteers were not taking medications, and 1-day prior to the game, they did not use deodorants, face or body creams neither perfume nor after shave.

Prior to starting the exercise, the volunteers meticulously cleansed the collection area, the forehead and face skin zones, with gauze soaked with isopropanol (LABCHEM®) in distilled water to avoid sample contamination.

Samples were collected in sterile 2 ml clean glass vials (9-425 C0000752) with screw cap and red PTFE/white silicone septa (Alwsci® Technologies). Sampling was achieved at a period of 5 min after training. A vial was leaned in the skin sampling zones. The sweat drops were collected by letting the drops flow naturally to the vials. It was collected one vial per volunteer, and the volume collection of each was approximately 200 μl.

### Samples Storage

Individual samples of volunteers were transported to the laboratory in refrigerated bags between 2 °C and 6 °C. These were immediately stored frozen at − 20 °C and analysed after 24 h.

### Sample Preparation for Analysis

Extraction of sweat for analysis by LC-MSMS using a electrospray ionization source (ESI) and multiple selected reaction monitoring mass spectrometry (MRM-MS) analysis was attained using a pool sample of eccrine sweat of healthy controls of young male volunteers (ages between 22 and 26) after physical training.

#### Sample Pool Preparation

After 24 h of collection the frozen samples from each vial were thawed and vortexed (Combi-Spin, FVL-2400 N, Biosan®) for 15 s. Three pooled samples were prepared taking 50 μl aliquots of all the volunteers. The procedure for the analytical profiling detection and identification by LC-MSMS was the same for all the pooled samples.

#### Sample Preparation for LC-MSMS Analysis

All plastic material and glassware were cleaned carefully to avoid contamination. Organic solvents (LC-MS grade) and distilled water were evaluated before use to minimize background interferences.

The liquid-liquid extraction was performed with 250 μl of pooled sample and 250 μl methanol LC-MS grade in a sterile 2 ml vial (Alwsci®) with screw cap and red PTFE/white silicone septa. The extract was centrifuged for 10 min at 3000 rpm using a Sigma 3 K30 centrifuge from B. Braun Biotech International GmbH. The supernatant liquid extract was transferred using a 500 μl syringe (Gastight 1750 Hamilton®) and filtered with a 13-mm, 0.22-μm nylon syringe filter (Filter-Lab®) into a conical insert (5.8 x 31.5 mm for 2 ml-N2004) into a sterile 2 ml vial (9-425 C0000752) with screw cap and red PTFE/white silicone septa (Alwsci®). The sample is successively analysed after preparation and stored in the LC sampler at 6 °C for LC-MSMS analysis.

### Instrumental

The LC-MSMS analysis was performed using a Dionex® Ultimate 3000 System UHPLC^+^ focused and a TSQ Quantis^TM^ triple-stage quadrupole mass spectrometer (Thermo Scientific, Waltham, MA).

The liquid chromatograph, an ultra-performance liquid chromatograph (UHPLC), was equipped with four modules, a SR-3000 Solvent Rack, a LPG-3400RS pump, an WPS-3000TRS auto sampler with temperature control and a TCC-3000RS column compartment from Thermo Scientific Dionex Ultimate 3000 series UHLPC^+^ focused.

The triple-stage quadrupole mass spectrometer was equipped with an electrospray ionisation (ESI) source.

The TSQ Quantis Mass Spectrometer is controlled by the TSQ Quantis 3.1 Tune software (Application 3.1.2415.15 Thermo Scientific), and the LC-MSMS operation and acquisition data system is controlled by the XCaliburTM 4.1 Thermo Scientific SP1 (0388-00CD-7B33) software.

### Chromatographic and Mass Spectrometry Conditions

The sample injection volume was 10 μl. The separation of biomarkers was achieved using an Accurore^TM^ RP-MS Column (2.6 μm, 150 × 2.1 mm, ThermoFisher Scientific). The gradient mobile phase consisted of water with 0.1% formic acid (A) and acetonitrile (B).

Mass spectrometry (MS) analysis was carried out using the triple-stage quadrupole mass spectrometer. The pooled samples were first injected in full-scan acquisition mode in positive and negative ion spray voltage acquisition modes (two times) to obtain information on the product ion of the most relevant compounds. Table 1 outlines the instrumental parameter settings used for chromatographic and mass spectrometry conditions.

**Table 1:** LC-MSMS operational conditions

LC	
UHPLC pre-column UHPLC column	Security Guard^TM^ Ultra Holder (AJO-9000 phenomenex®) Accurore^TM^ RP-MS Column (2.6 μm, 150 × 2.1 mm, ThermoFisher Scientific)
Column temperature	25 °C
Flow rate	0.25 ml min^-1^
Mobile phase	(A) H_2_O: 0.1% formic acid (V/V) (B) Acetonitrile Equilibration: B: 20% (5 min) Elution B: 20% (0 min); 90% (1 to 6 min); 80% (7 min); 50% (7.5 min); 20% (8 to 20 min)
Injection volume	10 μl
**MSMS**	
Ionization Source Analysis mode Ion-spray voltage Vaporizer temperature Capillary temperature	ESI positive and/or negative Full scan (Q3) and selective reaction monitoring (SRM) Positive (3500 V) and negative (3500 V) 320 °C 325 °C

After detection/identification by full-scan analysis, the small target biological molecules have been detected, NTs (monoamines, amino acids, acetylcholine, adenosine and other molecules (amino acids precursors of biogenic amines, amino acids, carboxylic acids, carbohydrates, breakdown product and steroid hormones). Optimization of analytical parameters for MSMS detection for some of these molecules indicated in 2.1.1 section was performed using analytical standards. The other biomarkers have been identified using the analytical conditions pre-defined by the MSMS software equipment, TSQ Quantum 3.1 Tune software. Samples were then injected in select reaction monitoring (SRM) mode, using Multiple Reaction Monitoring (MRM). Two selective MRM transitions were monitored for each targeted analyte according to Commission Decision Directive [26] for identification confirmation. These results are presented in Table 2.

**Table 2:** Identified biomarkers. Biomarker, retention time (rt), ESI operation mode, precursor ion (*m*/*Z*) and product fragment ions (m/z), signal (NL), collision energy (eV) and references for identification (Ref.)

Biomarker	rt	Mode	Precursor ion (***m***/***Z***)	Fragment ions (***m***/***Z***)	Signal	Collision energy (eV)	Ref
**Major NTs**							
Ach	1.59	+	146	60/87	10^3^	10/19	[24]
**Biological amines and metabolites**							
DA	1.59	+	154	91/137	10^2^	4	[24, 25]
DOPAC (DA Met)	2.34	-	167	122	10^2^	10	[24, 25]
3-MT (DA Met)	1.59	+	168	121	10^4^	10	[24, 25]
HVA (DA Met)	2.69	-	181	122	10^2^	10	[24, 25]
E	1.59	+	184.1	106/166	10^3^	12/21	[24, 25]
NE	2.56	+	170	107/135	10^2^	24/15	St
5-HT	1.37	+	177	115/160	10^3^	4	[24, 25]
5-HIAA (5-HT Met)	1.58	-	190	146	10^3^	13	[24, 25]
**Amino acids**							
Glu	1.57	+	148	84/130	10^5^	10	[17, 27]
**Purines**							
Ade	1.59	+	268	136/170	10^3^	12	[28]
**Other biomarkers**							
**Amino acid precursors of biological amines**							
Phe	1.59	+	166.1	77/103/120/149	10^7^	10	St, [17]
Trp	1.64	+	205.2	113/144/159/188/245	10^6^	10	St, [17]
Tyr	1.59	+	182	105/119/123/136/165	10^6^	10	St, [17]
**Amino acids**							
Crea	1.59	+	132.1	43.3/90.2	10^4^	10	[29]
Gln	1.32	+	147	84/85/103/121/130	10^5^	10	[17]
His	1.32	+	156	56/83/93/95/110	10^5^	10	St, [17]
Ile	1.59	+	132	69	10^5^	10	[28]
Leu	1.59	+	132.1	86	10^7^	10	[17]
Lys	1.05	+	147.2	56/84/130	10^5^	10	St
**Carboxylic acids**							
Asc	1.99	-	175	87/115	10^3^	23/15	St, [30]
Lacta	1.58	-	89	43	10^6^	10	[27, 31]
**Carbohydrates**							
Gluc	1.58	-	179	71/89	10^5^	10	[32]
**Breakdown product**							
Creat	1.58	+	114	44.3/86	10^3^	10	[29]
**Steroid hormones**							
Cor	1.59	+	363.1	121/309/327	10^4^	10	[11, 23]
Cort	1.59	+	361	163/343	10^4^	10	[23]

## Results

The results obtained from the sweat pool attained from 10 healthy young male volunteers after a football game (university students aged between 22 and 26, that typically play one 90-min football game per weak) are listed in Table 2. From the identified molecules, some already described stress-related biomarkers were found such as epinephrine or cortisol. Potential identified biomarkers are major NTs and other molecules, listed below.

Potential identified biomarkers:

- *NTs*: acetylcholine (Ach); biological amines and their metabolites, dopamine (DA), 3,4-dihydroxyphenylacetic acid (DOPAC, DA metabolite), homovanillic acid (HVA, DA metabolite), 3-methoxytyramine (3-MT, DA metabolite), epinephrine (E), norepinephrine (NE), serotonin (5-HT, ) and 5-hydroxyindol-3-acetic acid (5-HIAA, 5-HT metabolite); amino acids, glutamic acid (Glu); purines, adenosine (Ade)
- *Other identified molecules:* Amino acid precursors of biogenic amines, L-phenylalanine (Phe), L-tyrosine (Tyr) and L-tryptophan (Trp); amino acids, creatine (Crea), L-glutamine (Gln), L-histidine (His), L-isoleucine (Ile), L-leucine (Leu) and L-lysine (Lys); carboxylic acids, ascorbic acid (Asc) and lactic acid (Lacta); carbohydrates, D-glucose (Gluc); breakdown products, creatinine (Creat); steroid hormones, cortisol (or hydrocortisone) (Cor) and cortisone (Cort).

**Fig. 1:**
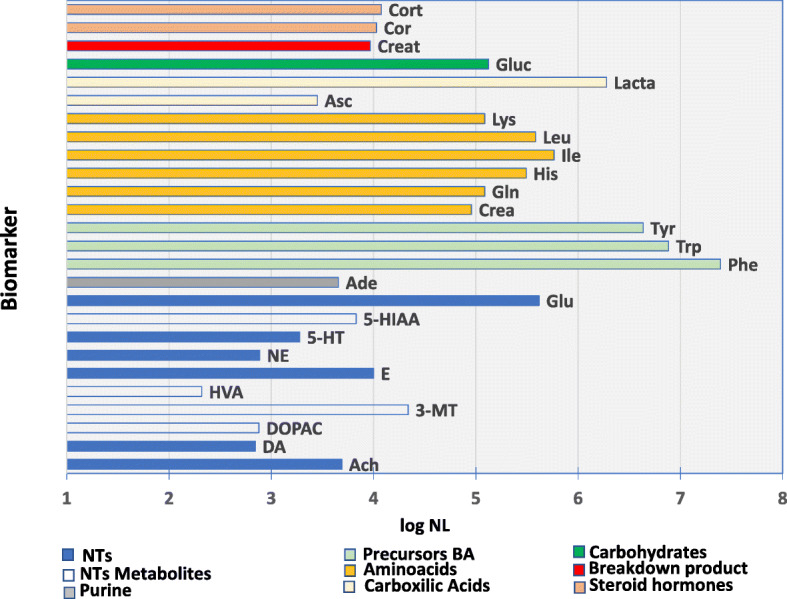
Representation of the identified biomarkers in function of log NL signal

Alternation of ESI polarity during the analysis enabled detection. Vaporizer temperatures were tested; collision energy and fragmentation were optimised. The selection of the analytical MSMS conditions was function of all the molecules to confirm the identification in biological samples, trying to obtain the best analytical compromise. The experimental signal described by the normalization level (NL), which describes the intensity of the base peak results, was the criteria to decide about the biomarkers detection. As so, compounds detected and identified with NL signals, between 10^2^ and 10^3^, 10^3^ and 10^4^ and higher than 10^4^ are considered as low intensity, medium intensity and high intensity biomarkers, respectively. As indicated in Table 2, represented in Fig. 1 and relating the identified biomarkers with the NL signal, it is possible to have the classification of biomarkers accordingly as follows:

- Low NL signal: DA, DOPAC, HVA and NE;
- Medium NL signal: Ach, E, 5-HT, 5-HIAA, Ade, Asc and Creat;
- High NL signal: 3-MT, Glu, Phe, Trp, Tyr, Crea, Gln, His, Ile, Leu, Lys, Lacta, Gluc, Cor and Cort.

Original experimental LC-MSMS chromatograms and MSMS spectra confirming the biomarker identification are supplied as supplementary material (Supp. Material, Table A).

*St* analytical standard analysed by LC-MSMS

## Discussion

The restricted amount of volume and/or biomarker abundance in each individual sample is a limiting factor for the detection. Thus, to explore the profile of stress biomarkers related with physical training in sweat samples, the pooling design was considered more efficient than a random sample strategy since it provides greater abundances that enhance the ability to identify and quantify more biomarkers. Before the methodology development and optimization for biomarker identification in the sweat samples, it was necessary to consider the chemical properties of the target biological molecules. The previous research on the function of the eccrine gland [20], on the artificial sweat composition [33] and on the information about stress biomarkers related with exercise [11, 14–16, 18, 19, 21, 22] were the default knowledge of the state of the art for the decision making for selecting the initial biomarkers to be detected in a sweat profile. Taking in consideration the literature available data, the chemical properties and the more recent available techniques [14, 16, 17, 21, 24, 25, 34], neurotransmitter (NT) biomarkers and other biomarkers that may be related with stress in physical training and/or associated with renal, liver and heart health functions [16, 22, 27] were considered as the primary target molecules for identification.

Also, at the extraction step, it was necessary to balance the chemical properties of the most likely target biomarkers to identify, with the objective to obtain a profiling screening with the detection by LC-MSMS of the maximum number of potential biomarker compounds in the samples, despite of the analytical recovery, which was not a concern at this stage.

The chromatographic optimization studies were primarily focused in finding a stationary phase able to retain the analyte employing suitable mobile phases. UHPLC, using a principle like high pressure liquid chromatography (HPLC), yields a significant improvement in the rapid separation of analyte via its smaller particle-size stationary phase and ultra-high-pressure pumps. It is therefore better suited for NT separation than conventional HPLC [16].

In the sweat sample clean up preparation for analysis, salts and proteins contained in the samples are not appropriate for ESI source causing signal suppression. For instance, the sensitivity of ESI is not favoured by highly polar and small molecules, such as NTs [16, 35] that are more susceptible to ion suppression. These considerations highlight the importance of sample pre-treatment prior analysis. Owing to the wide-ranging efficiency and time consumption of derivatization, a simplified sample preparation using LC coupled with ESI tandem mass spectrometry was employed for the biomarker identification in the sweat samples.

The above summarized considerations led to the selection of a simple liquid-liquid extraction with methanol by centrifugation with precipitation and filtration of supernatant with nylon filters 0.20 μm for sample clean-up prior to detection analysis. As mentioned earlier, the first analysis by LC-MSMS was performed in full scan in ESI-positive operation mode and in full scan in ESI-negative operation mode for selection and identification of biomarkers (analytical conditions indicated in Table 1).

The most relevant stress biomarker compounds preliminarily detected, after full-scan analysis, were selected for posterior SRM mode, using MRM analysis. The identification and confirmation of the compounds were performed according to the procedure prescribed by Commission Decision [26].

The major focus on biomarkers was towards neurotransmitters and other molecules that are considered (or may be related) as stress biomarkers associated with sports physical training [1, 3–5, 11, 15, 19].

The confirmation of the molecule identification was attained using bibliographic research and/or the analytical reference standards for experimental MSMS conditions as indicated in Table 2.

The tandem mass spectrometry conditions were first selected through bibliographic research [11, 17, 23, 25, 27–33]. The analytical standard analyte of E, NE, Phe, Trp, Tyr, His, Lys and Asc was used to confirm the biomarker identification (these have certified content and purity that is used as a reference in the analysis).

The quantification (Fig. 1) of low intensity biomarkers will require the further optimization of the analytical extraction conditions and/or its derivatization aiming to increase the mass, by introducing an electrophilic group and enhancing chromatographic separation (bib DA). This means that for the quantification step, the analytical extraction will not be the same for all the biomarker molecules. Alternatively, instead of the primary biomarker detection, it is possible to detect one or more of its metabolites as target molecules, e.g. as the case of DA that degrades into 3-MT which, by presenting higher NL, allows to use the same extraction method for quantification, attaining the same profiling screening. Often, metabolites turnover are in fact a more reliable assessment of the metabolic activity than the primary compound absolute concentration [25].

Evaluation of the potential stress biomarker profile provides extensive sweat composition data. In the present study, 26 potential biomarkers were identified. The identified exercise-related biomarkers suitable for future studies are listed below with reference of their major health function.

### Neurotransmitters (NTs)

- *Monoamines (DA, 5-HT, NE, E) and/or its metabolites.* These act via dopaminergic and adrenergic receptors, taking part in the regulation of the stress response, psychomotor activity, emotional processes, learning, sleep and memory [25]. The contribution of these NTs in multiple regulatory systems and metabolic processes supports their importance as biomarkers for the diagnosis, therapy and prognosis of several neuroendocrine and cardiovascular disorders [16].
- *Amino Acids (Glu).* **Glu** is one of the most abundant-free amino acid in the brain and is classified as excitatory NT [16]. Amino acid NTs represent a major class of biochemical compounds involved in neuronal communications at synapses in the central nervous system.
- *Ach.* One of the NTs released from cholinergic neurons in the central nervous system (CNS), plays an important role in sleep regulation, learning and memory, cognitive functions, and pathology of neurological disorders; a decrease in Ach levels in the brain is well established as a contributor to memory dysfunction in Alzheimer’s disease [36].
- *Ade.* Has far-reaching effects as an extracellular signalling molecule inducing vasodilation in most vascular beds, regulating activity in the sympathetic nervous system, having antithrombotic properties and reducing blood pressure and heart rate [37]. For example, Ade is used for stress testing and induction of systemic (and coronary) hyperemia.

### Other Biomarkers Molecules Related with Physical Training

- *Amino acid precursors of biogenic amines (Phe, Trp and Tyr).* Phe is a metabolic precursor of Tyr. Trp is a metabolic precursor of 5-HT. Tyr is a metabolic precursor of dopamine. The amino acids Phe, Trp and Tyr are monoamine precursors related with neurotransmitter release [38].
- *Amino acids (Crea, Gln, His, Ile, Leu and Lys).* These are compounds that can be related with biomarkers of renal, liver and heart health [27, 28].
- *Carboxylic acids (Asc and Lacta)*. Asc (or vitamin C) is a water-soluble compound that possesses diverse functions in the body [39], such as an antioxidant, or, for example, being part of the immune system response, playing a role in cardiovascular disease [39–41]. Lacta has been demonstrated to be a potential indicator of chronic obstructive pulmonary disease and other lung diseases [31].
- *Carbohydrates (Gluc).* Metabolic homeostasis is tightly linked to innate immune and stress responses of Gluc [42, 43].
- *Breakdown product (Creat).* Breakdown product of renal function and most widely used as biomarker for renal function [27, 29].
- *Steroid hormones (Cor and Cort).* Cor has long been recognized as the “stress biomarker” in evaluating stress related disorders [10] and is secreted by the adrenal glands mainly in response to stress. Cort is the inactive precursor of Cor. Cor is crucial for homeostatic maintenance, by means of modulating, regulating or influencing vital systems including neural, immune, cardiovascular, metabolic and endocrine systems. Prolonged elevated levels can cause impaired cognitive performance, hyperglycaemia, sleep disruption, elevated blood pressure, suppressed immune function, obesity and fatigue [11].

The obtained screening using LC-MSMS in MRM-MS mode is a viable analytical strategy for the discovery/identification of potential sweat stress biomarkers related with sports training.

Potential applications of these results may consider training sports where it remains necessary to have biomarkers for assessment and measurements of sports training induced stress that may lead to injuries. As an example, in football and other sports, stress may affect the sportsman health and performance. Muscle injuries can occur as an effect of immune response to stress [4, 5], and also, heart attack and kidney failure were already reported as a stress consequence [1, 3–5].

These results clearly show the necessity of more detailed studies regarding individuals and pooled sampling and also with direct and indirect sampling devices, being one of the possible studies between vials and patches’ sampling comparison. Also, rest and exercise sweat sampling composition must be compared to point out the biomarkers selection regarding stress induced by exercise. For a proper sweat analysis, the analytical instruments and methods must be chosen accordingly with the target analyte. One of the main gains in using MSMS is the possibility to detect multiple biomarkers in one run. However, from the clinical point of view, it is still a costly and time-consuming technique. Some authors point the use of digital biomarkers as a possible route to overcome these disadvantages. The development of biosensors to assess biomarkers’ content variations could be another important route to achieve feasible and lower cost clinical diagnosis [44]. Much more work needs to be developed, namely the concept of normalization of sampled volume, since even for the cystic fibrosis this was still not achieved [18].

## Conclusions

The composition profile of pooled sweat samples after physical training corresponding to a 90-min football match provided the identification of 26 potential biomarkers by LC-MSMS. This identification is an important achievement for further studies and selection of most important biomarkers in sweat, not only for stress, but also for other possible conditions/pathologies or early diagnosis. Also, in this study, it was possible to show that NTs are possible to detect in the composition of sweat body fluid after exercise. Further developments of the project should clarify the differences between in rest and exercise conditions. The evaluation of sports performance is particularly relevant in training and competition, and the identified biomarkers may give a contribution for the assessment of athletes’ conditions through its sweat composition.

## Supplementary Information

**Additional file 1: Table A**. LC chromatograms and MSMS spectra for each biomarker indicating the retention time, the ESI operation mode, the precursor ion mass ratio and selected fragmentation(s) mass ratio(s).

## Data Availability

Data generated or analysed during this study are included in this published article [and its supplementary information files]. More details are available from the corresponding author on reasonable request.

## Abbreviations

Ach: Acetylcholine
Ade: Adenosine
Asc: Ascorbic acid
Cor: Cortisol (or hydrocortisone)
Cort: Cortisone
Crea: Creatine
Creat: Creatinine
DA: Dopamine
DOPAC (DA Met): 3,4-Dihydroxyphenylacetic acid (DA metabolite)
E: Epinephrine or adrenaline
ESI: Electrospray ionization
Gln: L-Glutamine
Glu: Glutamic acid
Gluc: D-Glucose
His: L-Histidine
5-HT: Serotonin
5-HIAA (5-HT Met): 5-Hydroxyindol-3-acetic acid (5-HIAA, 5-HT metabolite)
HVA (DA Met): Homovanillic acid (DA metabolite)
Ile: L-Isoleucine
Lacta: Lactic acid
LC: Liquid chromatography
LC-MSMS: Liquid chromatography with detection by tandem mass spectrometry
Leu: L-Leucine
Lys: L-Lysine
3-MT (DA Met): 3-Methoxytyramine (DA metabolite)
Met: Metabolite
MRM-MS: Multiple selected reaction monitoring mass spectrometry
MS: Mass spectrometry
MSMS: Tandem mass spectrometry
NE: Norepinephrine or noradrenaline
NL: Normalization level
NT: Neurotransmitter
NTs: Neurotransmitters
Phe: L-Phenylalanine
Ref: References for identification
rt: Retention time
SRM: Select reaction monitoring
St: Analytical standard analysed by LC-MSMS
Trp: L-Tryptophan
Tyr: L-Tyrosine
UHPLC: Ultra-high performance liquid chromatography
