# Supplementary material for: Screening of Potential Stress Biomarkers in Sweat Associated with Sports Training
Source: Sports Med Open. 2021 Jan 22;7:8. doi: 10.1186/s40798-020-00294-3 (PMC7822976; doi:10.1186/s40798-020-00294-3)
Supplement: Supplementary file 1 — Additional file 1: Table A. LC chromatograms and MSMS spectra for each biomarker indicating the retention time, the ESI operation mode, the precursor ion mass ratio and selected fragmentation(s) mass ratio(s). [file 40798_2020_294_MOESM1_ESM.docx]

**Supplementary material**

Table A. LC chromatograms and MSMS spectra for each biomarker indicating the retention time, the ESI operation mode, the precursor ion mass ratio and selected fragmentation(s) mass ratio(s).

| LC chromatogram/MSMS spectra |
| --- |
| ***Major NTs*** |
| Acetylcholine t_r_ = 1.59 +ESI SRM ms2 146.00 → 60.00/87.00 |
|  |
| ***Biological amines*** |
| Dopamine t_r_ = 1.59 +ESI SRM ms2 154.00 → 91.00/137.00 |
|  |
| DOPAC (DA Met) t_r_ = 2.34 -ESI SRM ms2 167.00 → 122.00 |
|  |
| 3-MT (DA Met) t_r_ = 1.59 +ESI SRM ms2 168.00 → 121.00 |
|  |
| HVA (DA Met) t_r_ = 2.69 -ESI SRM ms2 181.00 → 122.00 |
|  |
| Epinephrine t_r_ = 1.59 +ESI SRM ms2 184.10 → 106.00/166.00 |
|  |
| Norepinephrine t_r_ = 2.56 +ESI SRM ms2 170.00 → 107.00/135.00 |
|  |
| Serotonin (5-HT) t_r_ = 1.37 +ESI SRM ms2 177.00 → 115.00/160.00 |
|  |
| 5-HIAA (5-HT Met) t_r_ = 1.58 -ESI SRM ms2 190.00 → 146.00 |
|  |
| **Amino acids** |
| L-Glutamic Acid t_r_ = 1.57 +ESI SRM ms2 148.00 → 84.00/130.00 |
|  |
| **Purines** |
| Adenosine t_r_ = 1.59 +ESI SRM ms2 268.00 → 136.00/170.00 |
|  |
| **Other Biomarkers** |
| **Amino acid precursors of Biogenic Amines** |
| L-Phenylalanine t_r_ = 1.59 +ESI SRM ms2 166.10 → 77.00/103.00/120.00/149.00 |
|  |
| L-Tryptophan t_r_ = 1.64 +ESI SRM ms2 205.2 → 113.00/144.00/159.00/188.00/245.00 |
|  |
| L-Tyrosine t_r_ = 1.59 +ESI SRM ms2 182.00 → 105.00/119.00/123.00/136.00/165.00 |
|  |
| Ascorbic Acid t_r_ = 1.99 -ESI SRM ms2 175.00 → 87.00/115.00 |
|  |
| **Other biomarkers** |
| **Amino acids** |
| Creatine t_r_ = 1.59 +ESI SRM ms2 132.10 → 43.30/90.20 |
|  |
| L-Glutamine t_r_ = 1.32 +ESI SRM ms2 147.00 → 84.00/85.00/103.00/121.00/130.00 |
|  |
| L-Histidine t_r_ = 1.32 +ESI SRM ms2 156.00 → 56.00/83.00/93.00/95.00/110.00 |
|  |
| L-Isoleucine t_r_ = 1.59 +ESI SRM ms2 132.00 → 69.00 |
|  |
| L-Leucine t_r_ = 1.59 +ESI SRM ms2 132.10 → 86.00 |
|  |
| L-Lysine t_r_ = 1.05 +ESI SRM ms2 147.20 → 56.00/84.00/130.00 |
|  |
| **Carboxylic acids** |
| Ascorbic Acid t_r_ = 1.99 -ESI SRM ms2 175.00 → 87.00/115.00 |
|  |
| Lactic Acid t_r_ = 1.58 -ESI SRM ms2 89.00 → 43.00 |
|  |
| **Carbohydrates** |
| D-Glucose t_r_ = 1.58 -ESI SRM ms2 179.00 → 71.00/89.00 |
|  |
| **Breakdown product** |
| Creatinine t_r_ = 1.32 +ESI SRM ms2 114.00 → 44.30/86.00 |
|  |
| **Steroid hormones** |
| Cortisol t_r_ = 1.59 +ESI SRM ms2 363.10 → 121.00/309.00/327.00 |
|  |
| Cortisone t_r_ = 1.59 +ESI SRM ms2 361.00 → 163.00/343.00 |
|  |
